# Letter to the editor: blood processing and sample storage have negligible effects on methylation

**DOI:** 10.1186/s13148-018-0455-6

**Published:** 2018-02-14

**Authors:** Kira Groen, Rodney A. Lea, Vicki E. Maltby, Rodney J. Scott, Jeannette Lechner-Scott

**Affiliations:** 1 School of Medicine and Public Health, University of Newcastle, Callaghan, NSW 2308 Australia; 2 Centre for Information Based Medicine, Hunter Medical Research Institute, New Lambton Heights, NSW 2305 Australia; 3 Institute of Health and Biomedical Innovations, School of Biomedical Science, Queensland University of Technology, Kelvin Grove, QLD 4059 Australia; 4 School of Biomedical Sciences and Pharmacy, University of Newcastle, Callaghan, NSW 2308 Australia; 5 Division of Molecular Genetics, Pathology North, John Hunter Hospital, New Lambton Heights, NSW 2305 Australia; 6 Department of Neurology, John Hunter Hospital, New Lambton Heights, NSW 2305 Australia

**Keywords:** Methylation, Blood, Processing, Storage, DNA extraction

## Abstract

DNA methylation is a dynamic epigenetic mechanism. Researchers aiming to assess archived DNA samples are expressing concern about the effect of technical factors on methylation, as this may confound results. We reviewed recent reports examining this issue in blood samples and concluded that variation in collection, storage, and processing of blood DNA confers negligible effects on both global methylation and methylation status of specific genes. These results are concordant with studies that have investigated the effect of sample storage and processing on methylation in other tissues, such as tumour, sperm, and placenta samples.

## Introduction

DNA methylation can be thought of as punctuation for the genome, whereby the interpretation of genes can change via DNA-based markers that do not include variation in actual DNA sequence. For example, it has long been known that methylated CpGs at transcription start sites (TSSs) of genes can inhibit gene expression independent of any variation in the DNA sequence (e.g. single-nucleotide variants and copy number variants) [1]. Such mechanisms have been associated with a number of cancers [2, 3]. Similar to DNA sequence variants, methylation can be inherited across generations (e.g. imprinting) but is also dynamic and can be modified by environmental factors. The ability of methylation status to be moulded by different environmental exposures has provided the attractive notion that methylation acts as an interface between genes and the environment, accounting for complex disease susceptibility. Epigenome-wide association studies (EWASs) involve surveying DNA methylation status at CpGs across the entire genome in an effort to determine differences associated with complex disease outcomes. With the realisation that the genome-wide association study (GWAS) approach is hitting unsatisfactory limits in terms of explaining disease risk and pathology and the advent of array-based and next-generation sequencing-based (e.g. bisulphite sequencing) technologies at reasonable cost, many researchers are now embarking on EWAS. For EWAS, investigators are looking to make use of the very large patient DNA banks that have been collected to help understand the trait of interest. However, since methylation is dynamic, obvious questions arise about factors that may influence methylation change other than the main hypotheses and whether or not these factors need to be controlled for. While age, sex, cellular heterogeneity of blood cells, lifestyle, and medication use are known sources of biological variability in DNA methylation profiles [4], variation in the way DNA has been collected, processed, and stored is less well understood and needs to be addressed. The following summarises current evidence on this topic, focussing on blood samples. An overview of the referenced studies can be found in Table 1.

**Table 1:** Summary of referenced studies

Ref no.	Author	Year	Sample	Anticoagulant	Processing	DNA extraction	Methylation	Results
[5]	Hebels	2013	Fresh blood	Citrate, EDTA, heparin	Whole blood stored up to 24 h at room temperature; buffy coat frozen at − 80 °C or in liquid nitrogen	QIAamp Blood Mini Kit (QIAGEN)	Infinium HumanMethylation450 Bead Chip (Illumina)	No significant effect on methylation profiles
[6]	Shiwa	2016	Fresh blood	EDTA, heparin	Whole blood stored at 4 °C for up to 24 h or at − 80° for 7 days	Maxwell 16 Blood DNA Purification Kit, QIAGEN Autopure LS, Gentra Puregene Blood Kit, QIAamp DNA Blood Maxi Kit, QIAGEN FlexiGene DNA Kit	Infinium HumanMethylation450 Bead Chip (Illumina)	Variation in methylation profiles could be corrected by adjusting for cell-type composition
[7]	Bulla	2016	Fresh blood	EDTA	Stored up to 1 year at 4, − 20, and − 80 °C	DNeasy Blood and Tissue Kit (QIAGEN)	Epitect Methyl II PCR Array “Human Stress and Toxicity” (QIAGEN)	Storage conditions had little to no effect on methylation
[8]	Huang	2017	Fresh blood	Heparin	Stored up to 15 days at 24 °C	QIAamp Blood Mini Kit (QIAGEN)	Pyrosequencing and dot blotting assay (anti-5mC antibody)	Methylation altered when sample was stored for longer than 3 days (study did not adjust for differences in cell-type composition)
[9]	Staunstrup	2016	Archived dried blood spots	N/A	Filter cards stored for up to 16 years at − 20 °C	DNA extraction according to St Julien et al. (2013), PLoS One [13].	DNA immunoprecipitation coupled with next-generation sequencing and pyrosequencing	Methylation profiles from archived samples comparable to fresh material
[10]	Soriano-Tarraga	2013	Fresh blood	EDTA	N/A	Autopure LS (QIAGEN), Puregen TM (Gentra Systems), and Chemagic Magnetic Separation Module I (Chemagen)	Luminometric Methylation Assay (LUMA)	Different DNA extraction methods may introduce some bias in GDM (medians: 78.1%, 76.5%, and 75.1%)
[11]	Bundo	2012	Fresh blood	Information not available	N/A	Phenol-chloroform extraction	Infinium HumanMethylation450 Bead Chip (Illumina) and pyrosequencing	Amplification bias could be greatly reduced by averaging technical replicates

## Sample collection

### Anticoagulants

The use of different anticoagulants is unlikely to introduce any significant variation in methylation status. There were no differences in CpG methylation of blood collected into EDTA, heparin, and citrate [5]. These results were consistent with the comparison of multiple biobank protocols that used blood collected into sodium heparin and EDTA in a multivariate analysis [6]. Different anticoagulants (citrate, heparin, and EDTA) did not affect DNA yield or quality [5].

## Sample storage

### Duration and temperature (whole blood and buffy coat storage)

While DNA yields, which may influence method selection for methylation assays, appear to be negatively affected by sample storage at higher temperatures and by the act of freezing the sample, comparable methylation profiles have been obtained across a range of blood storage conditions [5–7].

#### DNA yield and quality

DNA yield from EDTA whole blood may be negatively affected by storage at room temperature and 4 °C for longer than 24 h [6, 7], as well as by freezing the sample prior to DNA extraction [7]. No differences in DNA yield or quality were observed between samples frozen over different durations or at different temperatures (− 20 and − 80 °C), including storage in liquid nitrogen [5, 7]. The addition of the DNA stabilising agent DNAgard Blood Solution (Biomatrica) improved DNA yields when added before storage (for samples stored at − 80 °C), or prior to thawing (for samples stored at − 80 and − 20 °C) [7]. Different storage and transport conditions did not affect DNA quality assessed by Nanodrop 2000 UV–Vis Spectrophotometer (Thermo Scientific) [6], gel electrophoresis [6], or Bioanalyzer (Agilent) [8]. Additionally, DNA yield and quality of biobank samples stored for 13–17 years were still comparable to fresh material [5]. One small study found a decrease in leukocyte DNA yield after storage at room temperature for 3.5 days, but acknowledged that this decrease was highly correlated to a reduction in leukocytes [8]. Thus, DNA yield per cell likely remained stable.

#### Methylation

EDTA whole blood (*n* = 8) analysed immediately, stored at − 20 °C, − 80 °C, or at room temperature (following the addition of DNAgard Blood Solution) for up to 1 year, showed less than 1% variation in DNA methylation across all conditions. DNA was extracted with the DNeasy Blood and Tissue Kit (Qiagen), and methylation was assessed across the 22 genes represented on the Epitect Methyl II PCR Array “Human Stress and Toxicity” (Qiagen). The addition of the DNA stabilising agent did not affect methylation [7]. Notably, this study showed that methylation status of limited genes was maintained, yet changes in methylation status of other genomic regions cannot be excluded [7]. Storage of whole blood and buffy coats for 24 h to several months at 15 to − 80 °C, or in liquid nitrogen, introduced minimal bias in DNA methylation that could largely be corrected for by cell-type composition adjustments in a multivariate study [5, 6]. Prior “bench time”, leaving whole blood samples at room temperature for up to 24 h prior to separation of individual blood components (plasma, buffy coat, and erythrocytes), was also assessed and revealed minimal (0.6%) variation in CpG methylation [5]. A small study (*n* = 10) by Huang et al. [8] showed a change in methylation at a specific site after whole blood storage for 7 days at room temperature, as well as a decrease in global DNA methylation (GDM) for different whole blood storage conditions. While this study highlights the need to exert caution when not working with fresh material, the authors did not account for differences in cell-type composition, despite acknowledging a decrease in total leukocytes after storing whole blood at room temperature and reduced temperature for 3 days [8].

Long-term storage (13–17 years) of blood collected into both citrate and EDTA and stored in both liquid nitrogen and at − 80 °C did not significantly affect methylation profiles despite evidence of some scatter. However, comparable samples from the same individual analysed immediately after collection were not available and variability could be of inter-individual origin [5]. Even DNA methylation (assessed by DNA immunoprecipitation coupled with next-generation sequencing, MeDIP-seq, Illumina) of dried blood spots stored at room temperature for up to 16 years was comparable to samples stored for 4 years, and freshly collected samples, highlighting the stability of the methylome throughout long-term storage [9]. The majority of identified differences were located in repetitive regions, which are known for genetic variability. Noting that dried blood spot cards were not from the same individual and individuals were not age- and sex-matched, differences are likely a result of inter-individual variability, rather than technical noise introduced by long-term storage [9].

## Processing

### Cell composition

As expected, cell-type composition affected whole blood and buffy coat GDM and should be adjusted for comparability. Comparing major Japanese biobank blood collection protocols, Shiwa et al. [6] determined that pre-analytical bias could be accounted for when adjusting methylation profiles determined by the Infinium HumanMethylation450 Bead Chip array (Illumina) (450K array) for cell-type composition. For adjustments, cell-type composition was determined by flow cytometry. Importantly, the study noted that storage of samples at 4 °C for 24 h affected buffy coat cell-type composition, decreasing lymphocyte and increasing granulocyte counts [6]. It is our opinion that cell composition should be determined soon after sample collection to obtain reliable results.

### DNA extraction

There was no remarkable difference between various DNA extraction methods (Maxwell16 Blood DNA Purification Kit, Promega; Autopure LS, Qiagen; Gentra Puregene Blood Kit, Qiagen; QIAamp DNA Blood Maxi Kit, Qiagen; FlexiGene DNA Kit, Qiagen) following cell-type composition adjustments for buffy coat and whole blood samples [6]. A study by Soriano-Tarraga et al. [10] identified some variance in GDM between different DNA extraction methods (Autopure LIS, Qiagen; Puregen TM, Gentra Systems; Chemagic Magnetic Separation Module I, Chemagen); however, this variation did not reach statistical significance (*n* = 9) [10] and was comparable to technical variation identified by Bulla et al. [7]. Significant differences in GDM assessed by luminometric methylation assay (LUMA) were found in a large cohort (*n* = 580) of individuals recruited from different control registers. Nonetheless, inter-individual variability was not accounted for and data was not adjusted for known confounders of DNA methylation, such as alcohol consumption and white blood cell counts [10]. A systematic review of studies using LUMA and whole blood samples also supports the idea of DNA extraction bias in GDM [11]. Nevertheless, this systematic review only considered a small number of studies (of low power) and age differences between participants of the individual studies was not accounted for [10]. Differences potentially introduced by the use of different DNA extraction methods were much smaller than inter-individual differences [6].

DNA yields affect methylation assay selection, yet low DNA yields from whole blood extractions may be overcome by separating out buffy coats prior to extraction [6].

### Bisulphite conversion and whole genome amplification

Many DNA methylation assays require bisulphite converted DNA, yet bisulphite conversion causes DNA degradation. Consequently, DNA amplification is required. As DNA methylation status is lost during standard DNA amplification, bisulphite conversion must precede amplification, potentially increasing the risk of bias. Using a multiple displacement whole genome amplification method, whole genome amplification caused a significant decrease in reliably detected methylation sites compared to unamplified bisulphite-converted DNA. Equally, lower input of amplified bisulphite-converted DNA (10 vs. 50 ng) also resulted in a decrease in methylation signal determined by the 450K array (Illumina). Results were validated by pyrosequencing of randomly selected genes, and correlation between the two methods was high (*R* = 0.921). Variation was greatest in genomic regions showing neither hyper- nor hypo-methylation; however, averaging of technical triplicates was able to greatly reduce amplification bias [11].

## Conclusions and future directions

While sample collection, storage temperature, and storage duration showed negligible effects on DNA methylation, variation in bisulphite conversion may alter methylation at some loci. It is therefore our opinion that highly standardised protocols including technical replicates and clearly defined parameters for each of the collection and processing steps constitute the best practices for robust and accurate DNA methylation studies. However, results obtained from differently stored and processed samples may also be acceptable, as long as differences in cell-type composition and technical variation are taken into account. Some variation is to be expected when performing any methylation assay, and care should be taken not to interpret technical variation as biological difference. Nonetheless, the discovery of highly accurate DNA methylation biomarkers (e.g. methylation age [12]) would not have been possible if sample processing or storage had a significant effect on methylation profiles. Critical parameters to be considered when performing any methylation assay are summarised in Table 2. Increased confidence in methylation results may be achieved through replication in other laboratories and centres.

**Table 2:** Critical parameters for epigenome-wide association studies

Parameter		How to address
Inter-individual variability	Sex	Appropriate inclusion and exclusion criteria; record information; statistical corrections
	Age	
	Diet and lifestyle	
	Alcohol consumption	
	Medication use	
Variability in the sample	Leukocyte counts and composition	Determine leukocyte counts and cell-composition at sample collection and adjust methylation data accordingly
Variability introduced through processing	Changes in leukocyte counts with prolonged storage of whole blood	Determine leukocyte counts and cell-composition immediately or as soon as possible after sample collection
	Efficiency of bisulphite conversion	Include commercially available standards in the conversion reaction to determine conversion efficiency and include technical replicates
	DNA integrity post-bisulphite conversion	Assess DNA integrity post-conversion with a multiplex PCR assay

*PCR* polymerase-chain reaction

As a number of the studies included in this letter used loci-specific methylation assays, the question whether some loci are more susceptible to variation introduced by processing and storage remains to be answered.

## Abbreviations

450K array: Infinium HumanMethylation450 Bead Chip array (Illumina)
DNA: Deoxyribonucleic acid
EDTA: Ethylenediaminetetraacetic acid
EWAS: Epigenome-wide association study
GDM: Global DNA methylation
GWAS: Genome-wide association study
LUMA: Luminometric methylation assay
PCR: Polymerase-chain reaction
TSS: Transcription start site
